# A New Preview of Antibiotic-resistant Spectrum in Community-acquired Pneumonia: A Cross-Sectional Study from Iraq

**DOI:** 10.2174/0118743064437974260211062059

**Published:** 2026-02-18

**Authors:** Marwan Majeed Ibrahim, Yusur Muhammad Said Thabit Al-Rawi

**Affiliations:** 1 Department of Medicine, College of Medicine, Mustansiriyah University, Baghdad, Iraq; 2 Department of Surgery, College of Medicine, Ibn Sina University of Medical and Pharmaceutical Sciences, Baghdad, Iraq

**Keywords:** Antibiotic resistance, Antimicrobial stewardship, Community-acquired pneumonia, *S. sanguinis* bacteria

## Abstract

**Introduction/Objectives:**

Community-acquired pneumonia (CAP) remains one of the leading sources of morbidity/mortality in the world. However, the increasing problem of antimicrobial resistance (AMR) aggravates the issue. This research intends to explore the prevalent levels of resistance to antibacterial drugs in bacteria isolated from patients in Iraq experiencing CAP.

**Methods:**

A cross-sectional study was conducted on 122 clinically and radiologically confirmed CAP patients at Al-Yarmouk Teaching Hospital, Baghdad, from May 2023 to June 2024. Sputum samples were evaluated microscopically and analyzed for culture and sensitivity using the VITEK^®^ 2 system.

**Results:**

Of 122 patients, 44 (36.1%) had positive bacterial cultures. *S. pneumoniae* and *S. sanguinis* bacteria, along with *S. aureus* and *P. aeruginosa*, stood out as the common ones, causing 18.2% each. *S. aureus* comprised 11.4%, followed by *P. aeruginosa*. *S. pneumoniae* and *S. sanguinis* showed 100% resistance to azithromycin, clindamycin, and ciprofloxacin. *S. aureus* was 60% & 20% resistant to vancomycin & linezolid. Extremely alarming was the near-total resistance of *Acinetobacter baumannii*.

**Discussion:**

This study provides the first detailed resistance profile for CAP pathogens in Iraq using modern automated diagnostics. The findings reveal alarmingly high levels of antibiotic resistance, including pan-resistant strains.

**Conclusion:**

The problem of antibiotic resistance in CAP in Iraq is very serious and rising because of the development of pan-resistant bacteria. Currently, the requirement exists for comprehensive antibiotic stewardship programs in the whole of Iraq, along with strict implementation of measures against the free sale of antibiotics.

## 1 INTRODUCTION

Community-acquired pneumonia (CAP) continues to rank among the primary sources of illnesses and mortality despite being globally recognized [1]. It continues to affect millions of people in the world [2]. Early detection of the causative organism, together with the administration of the relevant antibiotics, remains crucial for better outcomes. However, the increasing problem of anti-microbial resistance (AMR) now continues to make the problem worse in the context of CAP in many areas that lack regulation in antibiotic use [3-5].

Antimicrobial resistance in CAP is not a micro-biological problem-it is a clinical epidemiological problem at the intersection of healthcare policy and therapeutic stewardship. The misuse of broad-spectrum antibiotics both in hospitals and communities has resulted in multidrug-resistant pathogens changing the microbiologic spectrum of CAP and defying traditional treatment algorithms [6, 7]. The Iraqi Ministry of Health (MOH) reported a high prevalence of drug-resistant forms of common bacterial pathogens and pan-resistance to line antibiotics such as meropenem [8]. Antibiotic stewardship, in the form of correct dosing and avoiding the prescription of antibiotics without a culture or sensitivity report, has been implemented in Iraq as a step toward the more reasonable clinical utilization of antibiotics [9].

Common causative bacteria for CAP are *Streptococcus pneumoniae*, Haemophilus influenzae, and Moraxella catarrhalis. These bacteria usually manifest with common symptoms such as productive cough, pleuritic chest pain, and fever [4]. However, in certain instances, atypical bacteria such as Mycoplasma pneumoniae, Chlamydophila pneumoniae, and Legionella pneumophila, together with respiratory viruses like SARS-CoV-2, can cause the condition to present in more insidious ways, often accompanied by extrapulmonary complications, including non-lobar infiltrates [4].

However, the differentiation of the two in the body is very difficult, given the overlapping symptoms. There are also certain host-related factors being recognized today for the purpose of contributing towards the acquisition of the resistance-causing bacteria in the body in the event of CAP [10].

This study aims to characterize the current landscape of antibiotic resistance in CAP among Iraqi patients. Specifically, it seeks to identify the prevalent bacterial pathogens and their resistance profiles to guide clinical decision-making and support the development of more effective antimicrobial stewardship policies in Iraq.

## 2 MATERIALS AND METHODS

### 2.1 Study Setting

This study was conducted and reported in accordance with the Strengthening the Reporting of Observational Studies in Epidemiology (STROBE) guidelines for cross-sectional studies [11]. It is a cross-sectional study involving 122 patients with clinically and radiologically documented cases of CAP collected from the medical ward, emergency department, and outpatient clinic in AL Yarmouk teaching hospital from May 2023 to June 2024.

### 2.2 Patients and Data Collection

All the cases that had been diagnosed as pneumonia, depending on the treating physician's notes and radiologist confirmation on corresponding images, had been sent for sputum evaluation with bacterial culture and sensitivity after evaluation of sputum under a microscope for detection of eligibility criteria for an acceptable specimen; more than 25 leukocytes and fewer than 10 epithelial cells per low power field. All specimens of patients were collected and immediately sent to the Laboratory Department, then evaluated using the VITEK^®^ 2 system to identify CAP-causing bacteria and antibiotic susceptibility.

Exclusion criteria included patients with active malignancies on chemotherapeutic agents, rheumato-logical illnesses on biological agents or disease-modifying agents, patients with advanced systemic illnesses who had been nursed in the hospital or nursing facility, patients with active pulmonary tuberculosis or receiving treatment for latent tuberculosis, and patients with acute pulmonary edema or recent pulmonary embolism.

### 2.3 Identification of CAP Isolates

Sputum samples that met quality criteria under microscopic examination (*i.e.*, >25 leukocytes and <10 epithelial cells per low-power field) were processed immediately in the microbiology laboratory. Bacterial cultures were performed on common culture media (such as blood agar, chocolate agar, MacConkey agar) and incubated accordingly (aerobically or in 5% CO_2_ for 24-48 hours). Bacterial Colony characterization was done by VITEK 2 Compact. This system employs bacteriochemical characterization. Side-by-side antimicrobial sensitivity was also performed by VITEK 2 Compact. S SUSCEPTIBILITY was evaluated according to CLSI.

Sputum specimens were microscopically screened, and only those of good quality were included. Out of a total of 124 collected sputum specimens, 18 were rejected because of poor quality.

Antimicrobial susceptibility testing according to the VITEK 2 system and resistance interpretation from the CLSI M100 guidelines, 30th edition (2020), were used. All tested agents, including linezolid and moxifloxacin as newer drugs, were subjected to breakpoints based on these guidelines.

### 2.4 Laboratory Procedures and Quality Control

All sputum specimens were transported in sterile, leak-proof containers and processed within 30 minutes of collection to maintain sample integrity. Microbiological work was performed under standard Biosafety Level-2 (BSL-2) conditions. Bacterial identification and antimicrobial susceptibility testing were performed using the VITEK^®^ 2 Compact system. The following AST cards were used: AST-P580 for Gram-positive organisms and AST-N255 for Gram-negative isolates. Internal system controls were applied automatically with each run, and quality-control procedures followed CLSI recommen-dations using the reference strains *E. coli ATCC* 25922, *S. aureus* ATCC 29213, and *P. aeruginosa* ATCC 27853, tested weekly to ensure accuracy of performance.

Multidrug resistance (MDR) was defined according to the ECDC/CDC criteria as resistance to at least one drug in three or more antimicrobial classes [12]. To ensure unbiased selection of cases despite the consecutive sampling approach used in the study, all hospitalizations for community-acquired cases who could provide their own sputum samples for analysis were included. Despite the reduced selection bias in the consecutive sampling strategy used in the study, patients who could not produce their own sputum samples tended to be excluded.

### 2.5 Statistical Analysis

All collected data were coded and entered into IBM SPSS Statistics version 29 (Chicago, IL, USA). Descriptive statistics were used to summarize frequencies, percent-ages, means, standard deviations, and ranges. Logistic regression analysis was performed to explore potential predictors of multidrug resistance (MDR). The model outputs included odds ratios (ORs), 95% confidence intervals (CIs), and exact *p*-values. Given the exploratory nature of the study, no formal correction for multiple comparisons was applied.

There was no power calculation because the research was descriptive in nature. Variables for confounding, like comorbidities and previous antibiotic exposure, could not be controlled for because the data were not uniformly available in the patient files. This limitation is acknowledged. There are certain bacterial groups for which the number of isolates was 3–4.

To determine the significance of categorical values, the Pearson chi-square test was used. However, in instances where the expected count was small (less than 5), the Yates continuity correction or Fisher's exact test was used. To determine significance in the study, the significance level of 0.05 was used.

### 2.6 Ethical Approval

This study was approved by the Ethical Board of the Medical Department at Al-Yarmouk Teaching Hospital, in collaboration with the Internal Medicine Scientific Committee of the College of Medicine, Mustansiriyah University (IRB No: 22, Date: 3 April 2023). The research was conducted in accordance with institutional ethical standards and the principles outlined in the Declaration of Helsinki 1975, as revised in 2024. Written informed consent was obtained from all participants or their legal guardians after they were informed about the study’s objectives, methodology, potential risks, and benefits. All patient data were anonymized to ensure confidentiality and prevent identification.

## 3 RESULTS

### 3.1 Characteristics of the Patients

**Fig. (1):**
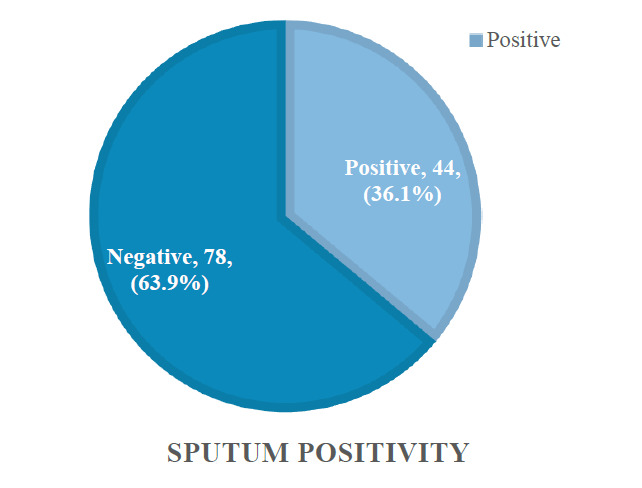
Flowchart of sputum culture positivity in the CAP cohort.

A total of 122 patients participated, of whom 44 patients had positive sputum culture detected (36.1%), and 78 patients had negative growth of any bacteria (63.9%) (Fig. **1**).

Of those 44 patients whose sputum culture was positive, 23 (52.3%) were male, and 21(47.7%) were female. The age distribution of those 44 patients was variable, and those aged 55 years old were 24 (54.5%).

Smoking history among those 44 patients was detected in 11 patients (25%), and 33 were nonsmokers (75%) (Table **1**).

### 3.2 Prevalence of CAP-causing Bacteria

Streptococcus pneumonia and *Streptococcus sanguinis* were the leading factors, accounting for 18.2% for each of all participants, followed by *Staphylococcus aureus* and Pseudomonas aeruginosa at 11.4%. The *Streptococcus mitis* accounted for 9.1% and *Staphylococcus epidermidis* for 9.1% too. Other bacteria form a minority in frequency, including Pseudomonas aeruginosa, Klebsiella spp. (oxytoca/pneumonia), and *Acinetobacter baumannii* were 6.8% each (Table **2**).

### 3.3 Antibiotic-resistant Prevalence

Starting with the streptococcus group, Streptococcus pneumonia exhibited a high prevalence of azithromycin resistance (100%), clindamycin resistance (100%), and ciprofloxacin resistance (100%), followed by doxycycline (88%) and ceftriaxone resistance of (88%), then levofloxacin (75%), then vancomycin resistance (63%), moxifloxacin seems to have intermediate resistance (50%), but linezolid still has a good susceptibility rate in which resistance only (12%).

Regarding *Streptococcus mitis*, it seems to be resistant to clindamycin, ciprofloxacin, levofloxacin, doxycycline, and azithromycin at an aerate of (100%), then moxifloxacin resistance (75%).

*Streptococcus sanguinis* exhibited a high prevalence of azithromycin resistance (100%), clindamycin resistance (100%), and ciprofloxacin resistance (100%), followed by doxycycline (100%) and ceftriaxone resistance (100%), then levofloxacin (88%) and moxifloxacin (63%).

Regarding *staphylococcus aureus* all 5 cases are resistant to ceftriaxone, doxycycline resistance was 80% as well as azithromycin, clindamycin resistance was 60%, ciprofloxacin and levofloxacin resistance were 40% and moxifloxacin found to have only one resistant case (20%); regarding anti staph antibiotics there’s 60% resistance rate to vancomycin and only one case found to have resistance to linezolid (20%).

Only four cases of *Staphylococcus epidermidis* were found, all of which were resistant to ceftriaxone (100%), clindamycin (75%), ciprofloxacin, and doxycycline (50%), azithromycin, moxifloxacin, and levofloxacin (50%). Only one case was found to be resistant to vancomycin (25%), and another case was found to have resistance to linezolid.

Regarding the Pseudomonas species, there were only three cases. Two cases were resistant to moxifloxacin, levofloxacin, ciprofloxacin, doxycycline, ceftriaxone, meropenem, and azithromycin (66%), and no case was resistant to piperacillin-tazobactam, ceftazidime, cefepime, or imipenem.

Klebsiella species were detected in only three cases. Two of the three cases were found to be resistant to moxifloxacin, levofloxacin, doxycycline, ceftriaxone, and azithromycin (66%), and only one of the three was resistant to ciprofloxacin, piperacillin-tazobactam, ceftazidime, cefepime, imipenem, and meropenem (33%).

Three patients were discovered to have *Acinetobacter baumannii* growth, and they were resistant to all antibiotics except one, which was sensitive to ciprofloxacin.

*Enterococci* growths were detected in 4 cases, and all were resistant to levofloxacin, doxycycline, and azithromycin (100%). Regarding ceftriaxone, ceftazidime, cefepime, piperacillin-tazobactam, imipenem, meropenem, and aminoglycoside resistance was 75%, and ciprofloxacin resistance was 50% (Tables **3****-****5**).

**Fig. (2):**
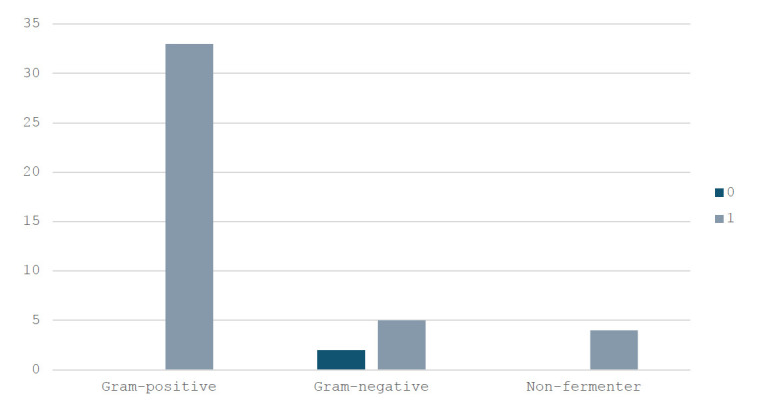
Distribution of multidrug-resistant isolates among gram-positive, gram-negative, and non-fermenter bacteria.

When stratified by bacterial category, Gram-positive organisms accounted for the majority of isolates with MDR (Fig. **2**).

### 3.4 Multivariate Analysis of Risk Factors for MDR Infection

To explore potential predictors of multidrug resistance, a binomial logistic regression was conducted with MDR status as the dependent variable. Age was included as a covariate, while gender, smoking status, and bacterial group were treated as categorical factors. The model demonstrated acceptable fit (McFadden’s R^2^ = 0.299), but none of the variables showed statistically significant associations with MDR (*p* > 0.05).

## 4 DISCUSSION

This paper discusses a common and underestimated problem in the management of pneumonia cases, mainly CAP, which is the increasing emergence of antibiotic resistance. The spectrum of antibiotic sensitivity and resistance is widely variable among countries, so there is no unified agreement between scientific societies for empirical antibiotic therapy in cases of CAP. The current research uses the VITEK 2 auto-analysis system. Also, the detection of pan-resistant Acinetobacter baumanii in association with high-level resistance in viridans group streptococci (*S. sanguinis* & S. mitis) in the context of CAP has not been previously reported in Iraq. Also, the current research work presents some updated data from 2023 onward, considering the rising shift in antibiotic availability in the region. Additionally, the scope of the research work is expanded by considering both ER and outpatient cases in Iraq.

In this study, only 44 of 122 patients had bacteria detected by sputum culture, which is almost equal to usual figures worldwide, which refers to the fact that most cases of CAP were found to be negative for sputum culture of bacteria [4].

Regarding the most common bacteria detected in sputum culture, there were streptococci in 21 of 44, which matches the usual figures of bacterial isolation in cases of pneumonia worldwide [4].

Antibiotic resistance is high among cases of Streptococcus pneumonia, mainly for the commonly prescribed antibiotics like azithromycin and ceftriaxone due to frequent prescription of these two drugs empirical in supposed cases of CAP and due to over the counter (OTC) prescription policy of antibiotic in Iraq which may permit to use these drugs without doctor prescription in most occasions, but still there is good sensitivity to moxifloxacin and linezolid which are to some limit newer antibiotics and not commonly to be noted on OTC prescription. Regarding other Streptococcus species, the same pattern is noted as that of *Streptococcus pneumoniae*, with high resistance to azithromycin, cephalosporins, fluoroquinolones, and doxycycline, but there is good sensitivity to linezolid, possibly due to the low prescription rate.

*Staphylococcus* is one of the most common pathogens that can cause CAP and may poses a difficulty in treating pneumonia caused by this pathogen, the main cause of this problem is the emergence of strains that is resistant to certain anti-staphylococcus antibiotics; Methicillin-resistant *Staphylococcus aureus* (MRSA) and to a lower frequency Vancomycin-resistant *Staphylococcus aureus* (VRSA) usually forms the main strains of *Staphylococcus aureus* infections. With consideration of the resistance pattern, there is a 60% rate of resistance to vancomycin, which is of great significance for the emergence of the VRSA strain. Linezolid still has good efficacy, which may be due to its low prescription rate and its status as a newer-generation anti-staphylococcal antibiotic compared with vancomycin. Regarding Pseudomonas species, there is good sensitivity to anti-pseudomonal antibiotics, although meropenem showed 2 resistant cases, which may be due to the frequent use of meropenem in practice in Iraq.

The pan-resistance observed in *Acinetobacter baumannii* isolates in this study aligns with global trends and underscores its critical clinical relevance. E. baumannii is one of the ESKAPE microbes-the acronym stands for Enterococcus faecium, *Staphylococcus aureus*, Klebsiella pneumoniae, *Acinetobacter baumannii*, Pseudomonas aeruginosa, and Enterobacter-they are renowned for their ability to resist the action of normal antibiotic drugs. These bacteria pose significant pharmacological resistance due to their rapid development of resistance to many drugs [13]. Among the most worrying bacteria is carbapenem-resistant A. baumannii (CRAB), ranked by the World Health Organization (WHO) since 2018 on its top list of research targets for antibiotic development. Carbapenem resistance is frequently used as a surrogate marker for extensive resistance, as it is often associated with co-resistance to multiple other antimicrobial classes [14].

Local epidemiology for antimicrobial resistance in Iraq reveals the worrisome increase in the prevalence of multidrug resistance among the causative microorganisms. According to the 2022 report by Awayid *et al*. [15] on MRSA isolated from hospitals in Iraq, the resistance rates exceeded 90% for penicillin and erythromycin. Moreover, SCCmec III was the subtype that dominated in the region. Notably, ST239 was the prevalent clone. This suggests the endemic prevalence of hospital-acquired MRSA along with the genomic diversity in the clones. Also similar was the observation by Hasan *et al*. (2020) [16], according to whom the 95%+ resistance rate in *P. aeruginosa* isolated from Kirkuk was ominously high for broad-spectrum β-lactam drugs ampicillin, amoxicillin, and cefixime. It was partially susceptible to both imipenem and gentamicin. Presently, therefore, the choice of drugs for therapy in the local context of Iraq is limited.

Rates of CRAB vary across regions, but a rising trend has been reported in several Arab League countries, particularly in the Levant region-including Iraq, Jordan, Lebanon, the Palestinian territories, and Syria. The increasing prevalence of these extensively drug-resistant strains has catalyzed efforts to develop novel antimicrobial agents and innovative therapeutic strategies.

*Enterococci* resistance is still high mainly for anti-gram-negative antibiotics like third and fourth generations cephalosporines and fluoroquinolones, the increase in resistance reduces the options for useful treatment of CAP caused by MDR Gram-negative bacteria and makes them a large risk for management failure and a major worldwide public health problem, associated with high costs on health services, longer hospital stays and increased mortality [5].

Our findings align with previous Iraqi research that underscores the high burden of antimicrobial resistance in CAP. In a cross-sectional study by Jaaffar *et al*. (2019) [17], Klebsiella pneumoniae was the most commonly isolated pathogen in pneumonia cases (54%), followed by *Streptococcus pneumoniae* (26%), with both organisms exhibiting substantial resistance to first-line antibiotics, particularly piperacillin (93.06% resistance in K. pneumoniae) and erythromycin (71.43% resistance in *S. pneumoniae*). These resistance trends mirror our own results and highlight the continued circulation of resistant strains in community settings. Moreover, Al-Jumaili *et al*. (2024) [8] emphasized that empirical prescribing practices dominate in Iraqi hospitals, with only 9% of antibiotics prescribed based on culture results, and a high prevalence of multidrug-resistant organisms, including *P. aeruginosa* and *E. coli.* The findings of our study are supported by recent large-scale Iraqi surveillance data. Al-Fahad *et al*. (2024) [18] analyzed over 11,000 clinical records from seven provinces and confirmed a widespread distribution of multidrug-resistant Klebsiella pneumoniae and *Staphylococcus aureus*, particularly among adult patients, with ampicillin and oxacillin reported as highly resistant antibiotics; findings that align closely with the resistance patterns observed in our CAP isolates. Moreover, Alwash *et al*. (2021) [19] highlighted the strain-specific variability in antibiotic resistance among *S. aureus* isolates in Iraq, using spa-typing, and noted consistent resistance to amoxicillin and oxacillin across burn and wound-derived strains, echoing the challenge of empiric therapy in high-resistance settings.

Our results highlight the importance of using context-adapted antimicrobial stewardship (AMS) strategies in Iraq. According to the systematic review by Monmaturapoj *et al*. (2021) [20], the implementation of AMS programs through pharmacist-led education in hospital settings achieved better compliance with the use of antimicrobial agents in line with recommended guidelines without adversely affecting patient outcomes. Notably, the approach is crucial in low- and middle-income countries like Iraq, because pharmacists play a pivotal role in the implementation of AMS programs in the absence of infectious disease specialists. Complementarily, Lam *et al*. (2021) [21] emphasized the success of community-level AMS models in similar settings, advocating for prescriber education, public awareness, and the regulation of non-prescription antibiotic sales. These evidence-based approaches offer a dual-pathway, hospital- and community-based approach that can be tailored to Iraq’s health system to address rising antimicrobial resistance.

Another critical contributing factor for the observed high resistance rates in Iraq is the indiscriminate utilization of non-steroidal anti-inflammatory drugs (NSAIDs) in the initial management of fever. Fever is known to be an adaptive physiological response that works in the body’s favor by increasing the immunity of the organism, the functionality of leukocytes, and the bactericidal effect of the antimicrobial therapy being used. However, in the initial stages of the infection process, the induction of antipyretic therapy might decrease the effect of the body’s natural fever response. Various studies suggest that hyperthermia improves the response to antimicrobial therapy in the body [22-25].

## 5 LIMITATIONS

This study has several limitations. First, it was conducted in a single tertiary care center, which may limit the generalizability of the findings to other healthcare settings in Iraq. Second, the relatively small number of culture-positive cases may not fully represent the broader spectrum of bacterial pathogens and resistance patterns in the community. Third, only sputum samples were used for microbiological analysis; blood cultures, bronchoalveolar lavage, and other diagnostic modalities were not included, which may have led to the under-detection of invasive or atypical pathogens. Fourth, no molecular resistance mechanism analysis was performed, limiting the identification of specific genetic determinants of resistance. Fifth, viral co-infections and atypical respiratory pathogens were not assessed due to testing limitations. Finally, although the study spanned 14 months, seasonal variation in respiratory infection rates may have influenced the distribution and frequency of isolated pathogens, potentially affecting temporal trends in resistance.

## CONCLUSION

There is a high antibiotic resistance rate among cases of CAP in this study, which may be related to the increasing use of antibiotics and a low level of antibiotic stewardship in Iraq. There is an emergence of new strains of pan-resistant or nearly pan-resistant bacteria. We recommend stricter antibiotic stewardship in Iraq and complete control of antibiotic distribution from pharmacies, encouraging treating doctors to minimize antibiotic prescriptions in the community, and greater utilization of culture and sensitivity testing for precise antibiotic use.

## Figures and Tables

**Table 1:** General information of participants.

**Category**		**No**	**%**
**Age (years)**	<55years	20	45.5
	=>55years	24	54.5
	Mean ± SD (Range)	55.9±18.2 (17-88)	
**Gender**	Male	23	52.3
	Female	21	47.7
**Smoking**	Smoker	11	25.0
	Not	33	75.0

**Table 2:** Sputum culturing results and prevalence of CAP-causing bacteria.

**Type of Bacteria**	**No.**	**%**
*Streptococcus pneumoniae*	8	18.2
*Streptococcus sanguinis*	8	18.2
*Streptococcus mitis*	4	9.1
*Streptococcus gordonii*	1	2.3
*Staphylococcus aureus*	5	11.4
*Staphylococcus epidermidis*	4	9.1
*Pseudomonas aeruginosa*	3	6.8
*Acinetobacter baumannii*	3	6.8
*Klebsiella spp. (oxytoca/pneumoniae)*	3	6.8
*Enterococcus spp. (avium/gallinarum)*	3	6.8
*Escherichia coli*	1	2.3
*Aeromonas hydrophila*	1	2.3

**Table 3:** Antibiotic resistance profile of gram-positive bacterial isolates in CAP.

**Pathogen**	**Patients Number**	**Resistant Cases (%)**								
		**Moxi**	**Levo**	**Cipro**	**Doxy**	**Linz**	**Van**	**Clind**	**Ceftr**	**Azithromycin**
***Streptococcus pneumoniae***	8	4 (50%)	6 (75%)	8(100%)	7(88%)	1(12%)	5(63%)	8(100%)	7(88%)	8(100 %)
***Streptococcus mitis***	4	3(75%)	4(100%)	4(100%)	4(100%)	2(50%)	2(50%)	4(100%)	2(50%)	4(100%)
***Streptococcus sanguinis***	8	5(63%)	7(88%)	8(100%)	8(100%)	1(12%)	2(25%)	8(100%)	8(100%)	8(100%)
***Streptococcus gordonii***	1	1(100%)	1(100%)	1(100%)	1(100%)	0(0%)	0(0%)	1(100%)	1(100%)	1(100%)
***Staphylococcus aureus***	5	1(20%)	2(40%)	2(40%)	4(80%)	1(20%)	3(60%)	3(60%)	5(100%)	4(80%)
***Staphylococcus epidermidis***	4	2(50%)	2(50%)	3(75%)	3(75%)	0(0%)	1(25%)	3(75%)	4(100%)	2(50%)

**Table 4:** Antibiotic resistance profile of gram-negative bacterial isolates.

**Pathogen**	**Patients Number**	**Resistant Cases (%)**										
		**Moxi**	**Levo**	**Cipro**	**Doxy**	**Ceftr**	**Piper**	**Ceftz**	**Cefep**	**Imip**	**Mero**	**Azithromycin**
***Pseudomonas sp.***	3	2(66%)	2(66%)	2(66%)	2(66%)	2(66%)	0(0%)	0(0%)	0(0%)	0(0%)	2(66%)	2(66%)
***Klebsiella sp.***	3	2(66%)	2(66%)	1(33%)	2(66%)	2(66%)	1(33%)	1(33%)	1(33%)	1(33%)	1(33%)	2(66%)

**Table 5:** Antibiotic resistance profile of *acinetobacter baumannii* and enterococcus species.

**Pathogen**	**Patients Number**	**Resistant Cases (%)**											
		**Moxi**	**Levo**	**Cipro**	**Doxy**	**Ceftr**	**Piper**	**Ceftz**	**Cefep**	**Imip**	**Mero**	**Azithromycin**	**Amg**
***Acinetobacter baumannii***	3	3(100%)	2(66%)	3(100%)	3(100%)	3(100%)	3(100%)	3(100%)	3(100%)	3(100%)	3(100%)	3(100%)	3(100%)
***Enterococci***	4	4(100%)	4(100%)	2(50%)	4(100%)	3(75%)	3(75%)	3(75%)	3(75%)	3(75%)	3(75%)	4(100%)	3(75%)

## Data Availability

The data supporting the findings of this study are available from the corresponding author [M.M.I] upon reasonable request.

## LIST OF ABBREVIATIONS

CAP: Community-acquired Pneumonia
AMR: Antimicrobial Resistance

## STANDARDS OF REPORTING

STROBE guidelines were followed.
